# Cardioprotective Effects of Dietary Flaxseed Post-Infarction Are Associated with Changes in MicroRNA Expression

**DOI:** 10.3390/biom10091297

**Published:** 2020-09-08

**Authors:** Mihir Parikh, Branislav Kura, Kimberley A. O’Hara, Elena Dibrov, Thomas Netticadan, Jan Slezak, Grant N. Pierce

**Affiliations:** 1 Department of Physiology and Pathophysiology, Faculty of Health Sciences, University of Manitoba, Winnipeg, MB R3E 09J, Canada; mparikh@sbrc.ca (M.P.); kohara@sbrc.ca (K.A.O.); tnetticadan@sbrc.ca (T.N.); 2 Institute of Cardiovascular Sciences, St. Boniface Hospital Albrechtsen Research Centre, 351 Taché Avenue, Winnipeg, MB R2H 2A6, Canada; edibrov@sbrc.ca; 3 Canadian Centre for Agri-food Research in Health and Medicine (CCARM), St. Boniface Hospital Albrechtsen Research Centre, 351 Taché Avenue, Winnipeg, MB R2H 2A6, Canada; 4 Institute for Heart Research, Centre of Experimental Medicine, Slovak Academy of Sciences, 814 38 Bratislava, Slovakia; bkura@sbrc.ca (B.K.); jslezak@sbrc.ca (J.S.); 5 Agriculture and Agri-Food Canada, St. Boniface Hospital Albrechtsen Research Centre, 351 Taché Avenue, Winnipeg, MB R2H 2A6, Canada

**Keywords:** myocardial infarction, microRNA, diet, heart, flaxseed, alpha linolenic acid

## Abstract

MicroRNAs (miRNAs/miRs) such as miR-1, miR-133a, miR-133b, miR-135a, and miR-29b play a key role in many cardiac pathological remodeling processes, including apoptosis, fibrosis, and arrhythmias, after a myocardial infarction (MI). Dietary flaxseed has demonstrated a protective effect against an MI. The present study was carried out to test the hypothesis that dietary flaxseed supplementation before and after an MI regulates the expression of above-mentioned miRNAs to produce its cardioprotective effect. Animals were randomized after inducing MI by coronary artery ligation into: (a) sham MI with normal chow, (b) MI with normal chow, and (c–e) MI supplemented with either 10% milled flaxseed, or 4.4% flax oil enriched in alpha-linolenic acid (ALA), or 0.44% flax lignan secoisolariciresinol diglucoside. The feeding protocol consisted of 2 weeks before and 8 weeks after the surgery. Dietary flax oil supplementation selectively upregulated the cardiac expression of miR-133a, miR-135a, and miR-29b. The levels of collagen I expression were reduced in the flax oil group. We conclude that miR-133a, miR-135a, and miR-29b are sensitive to dietary flax oil, likely due to its rich ALA content. The cardioprotective effect of flaxseed in an MI could be due to modulation of these miRNAs.

## 1. Introduction

Cardiovascular disease (CVD) is a leading cause of morbidity and mortality, and according to the latest estimates by the Heart Disease and Stroke Statistics—2019 update, approximately 1 of every 3 deaths occur due to CVD. Ischemic heart disease is the major cause of mortality as 43.2% of cardiovascular deaths are attributed to it alone [1]. Ischemia results in acute cardiac damage through myocardial infarction (MI). Insufficient blood flow to the myocardium results in an MI, which, in turn, leads to inflammatory and fibrotic changes in both viable and infarcted myocardium and consequently leads to adverse cardiac remodeling and ventricular dysfunction [2]. Recent advancements in the understanding of molecular mechanisms of heart function have shone a light on the impact of endogenously encoded microRNAs (miRNAs) on cardiovascular health and disease [3]. In contrast to standard pharmacological drugs acting on single targets, miRNAs are capable of modulating several downstream pathways in parallel [4].

miRNAs are endogenous, non-coding, single-stranded RNAs of 22–26 nucleotides that regulate messenger RNA (mRNA) expression through posttranslational inhibition or degradation [5]. Several miRNAs have been shown to be involved in cardiac pathological processes such as acute MI, cardiac arrhythmias, and hypertrophy [6,7,8]. miRNAs can either promote or inhibit apoptosis of cardiomyocytes, they can modulate angiogenesis, alter cardiac regeneration and reprogram cardiac fibroblasts into cardiomyocytes [9]. These effects clearly support a relationship between altered miRNA expression and ischemic heart disease. Recent studies have highlighted the importance of miRNAs in the regulation of cardiac rhythm [9]. miRs such as miR-1, miR-133, miR-29, and miR-135 play important roles in cardiac remodeling and arrhythmias. The present study focused on examination of miR-1, miR-133a/b, miR-135a, and miR-29b due to their involvement in cardiac remodeling processes including inflammation, apoptosis, arrhythmias, and fibrosis. Furthermore, miR-1 and miR-133 are cardiac-specific markers [10] and two of the most abundant miRs in the heart [11]. Increased expression of miR-1 has been associated with arrhythmias after an MI [9]. Upregulation of miR-133 reduces apoptosis of cardiomyocytes [12], decreases the inflammatory response in myocardium after an MI [11], lowers incidences of arrhythmias [13], and improves cardiac reprogramming [14]. miR-29b is considered to be a master regulator of cardiac fibrosis [15,16,17]. Although miR-135a is less widely studied, strong evidence supports its role in arrhythmias and cardiac fibrosis after an MI [18,19].

A recent meta-analysis of 13 randomized controlled trials with a total of 127,477 participants demonstrated a significant reduction in the risk of MI, total CAD, and total CVD through supplementation of the diet with marine omega-3 polyunsaturated fatty acids (n-3 PUFAs) [20]. An alternative source of n-3 PUFAs is being investigated due to limiting physical and physiological concerns regarding n-3 PUFAs obtained from marine sources [21]. Flaxseed is a rich source of ALA, which is a plant-based n-3 PUFAs. In addition to the high content of ALA, flaxseed is also a good source of lipid-lowering dietary fiber and the antioxidant secoisolariciresinol diglucoside (SDG), which is a plant lignan [21]. Dietary flaxseed recently demonstrated several positive effects in a coronary artery ligation induced model of MI in rats. Flaxseed supplementation reduced the incidence of ventricular arrhythmias as well as prevented an excessive increase in infarct size, myocardial fibrosis, inflammation, and left ventricular dilation [22]. However, the mechanism responsible for this cardioprotective effect of flaxseed was unclear. In view of the above discussion of the impact of miRNAs on cardiac performance under pathological challenge, it is possible that dietary flaxseed may alter miRNA expression and, ultimately, affect myocardial adaptive processes. In support of this hypothesis, diet is known to induce significant cardioprotective effects via a direct action on the epigenome [23]. The current investigation was thus performed to test the hypothesis that flaxseed supplementation exerts its cardioprotective effect by modulating the expression of miR-1, miR-133a, miR-133b, miR-135a, and miR-29b, which are involved in the adverse cardiac remodeling process after an MI.

## 2. Materials and Methods

### 2.1. Animal Care

This study protocol (17-005) was approved by the University of Manitoba Office of Research Ethics & Compliance and Animal Care Committee and was done in accordance with the guidelines by the Canadian Council for Animal Care. Male Sprague Dawley rats (100–140 g; Charles River Laboratories, Quebec, Canada) were housed in a temperature and humidity-controlled room with a 12 h light/dark cycle. Animals were randomized into five groups: (a) sham MI fed normal chow, (b) MI fed normal chow, (c) MI fed chow supplemented with 10% milled flaxseed (flax), (d) MI fed chow supplemented with 4.4% flax oil enriched in alpha-linolenic acid (Oil), and (e) MI fed chow supplemented with the flax lignan secoisolariciresinol diglucoside (SDG) (0.44%). These dietary interventions and the concentrations used are similar to those used successfully in previous studies [24]. The amount of ALA present in 10% milled flaxseed is equivalent to the amount in 4.4% flax oil. The amount of SDG present in 10% milled flaxseed is equivalent to the amount in 0.44% flax lignan. The four diets included a Prolab^®^ RMH 3000 rodent regular chow (TestDiet, Richmond, IN, USA), a 10% ground flaxseed (BakePur Milled Golden Flaxseed, Pizzey Ingredients, Russell, MB, Canada) supplemented chow diet, or a 4.4% flax oil (Cold Pressed Flax Oil, Royal Harvest, Shape Foods, Brandon, MB, Canada) supplemented chow diet, or a 0.44% SDG (Archer Daniels Midland, Chicago, IL, USA) supplemented diet.

After receiving respective diets for two weeks, animals underwent either coronary artery ligation (CAL) or sham surgery. Animals were anesthetized with 1–5% isoflurane and oxygen at a flow rate of 2 L min^−1^ and kept in a surgical plane of anesthesia with 2% isoflurane during surgery. A left thoracotomy was carried out, and the heart was gently exposed from the pericardial sac through the incision. The left anterior descending coronary artery (LAD) was located and occluded with 6-0 polypropylene silk suture at about 2 mm from aortic root. The suture was tied, and the ligation was estimated to be successful when the anterior wall of the left ventricle turned pale. The heart was repositioned, then the chest compressed to remove any air from the cavity and the incision was closed using a purse string suture. Sham-operated animals that served as controls were subjected to similar surgical procedures except that the LAD artery was not ligated. Buprenorphine 0.05 mg kg^−1^ was administered pre- and post-surgery (2 times a day for 2 days) subcutaneously as an analgesic agent to all animals. Animals were fed their respective diets for two weeks before and for 8 weeks after surgery. Thus, animals were sacrificed after a total of 10 weeks on the study protocol.

### 2.2. Biological Sample Collection and Analysis

All animals were anesthetized with ketamine/xylazine (9.0 mg per 100 g/0.9 mg per 100 g IM). The depth of anesthesia was assessed by a pedal withdrawal reflex. The blood sample was collected from the inferior vena cava by opening the thoracic cavity and the heart was immediately excised. The whole heart was rinsed in phosphate buffered saline (PBS), and the left ventricular (LV), septum, and fibrotic scar tissues were separated, weighed, and flash-frozen in liquid nitrogen.

### 2.3. Dietary Fatty Acid Analysis

Total fatty acids were extracted from the diet samples and derivatized as previously described [22]. The fatty acid methyl esters were separated on a DB225MS column (30 m × 0.25 mm diameter and 0.25 μm film thickness; Agilent Technologies Canada Inc., Mississauga, Ontario) using a Varian 450 GC with FID. The temperature program was 70 °C for 2 min, the temperature was raised to 180 °C at 30 °C/min, held for 1 min, raised to 200 °C at 10 °C/min, held for 2 min, then raised to 220 °C at 2 °C/min and held for 10 min before finally raising to 240 °C at 20 °C/mine and holding for 15 min. Total run time was 46.67 min, and samples were run with a 20:1 split ratio and a 1.3 mL/min column flow. Hydrogen was used as the carrier gas. Components were identified by comparison with authentic standards (Nu-Chek Prep, Elysian, MN, USA).

### 2.4. Western Blot Analysis for Collagen 1A1 and the Na^+^-Ca^2+^ Exchanger (NCX)

The viable (non-infarcted) LV tissue samples were homogenized in RIPA buffer (Teknova, Hollister, CA, USA). The extracted protein was quantified using Pierce^TM^ BCA Protein Assay Kit (Thermo Scientific, Waltham, MA, USA). Equal amounts of protein samples were separated on 7.5% sodium dodecyl sulphate-polyacrylamide gels (SDS-PAGE) using TGX Stain-Free^TM^ FastCast^TM^ Acrylamide Kit (Bio-Rad, Hercules, CA, USA). After electrophoresis, the separate proteins were transferred using the Trans-Blot Turbo Mini Nitrocellulose Transfer Packs and the Trans-Blot Transfer System (Bio-Rad, Hercules, CA, USA). The membranes were blocked with 5% dry milk in Tris-buffered saline/0.1% Tween 20 (TBST) for 1 h at room temperature, and then incubated overnight at 4 °C with a primary anti-rat collagen type I (1:1000, CL50141AP, Cedarlane, Burlington, ON, Canada) or monoclonal anti-Na^+^-Ca^2+^ exchanger (1:1000, R3F1, Swant, Fribourg, Switzerland) in 1% dry milk. The membrane was washed with TBST and then incubated with secondary antibody IgG (1:10,000, Abcam, Cambridge, MA, USA) conjugated with horseradish peroxidase in 1% dry milk. The membrane blots were developed using Luminata^TM^ Forte Western HRP Substrate (MilliporeSigma, Burlington, MA, USA) and imaged on a Gel Doc^TM^ XR+ imager (Bio-Rad, Hercules, CA, USA).

### 2.5. MicroRNA Detection in Cardiac Tissue

Total RNA was isolated from the viable LV tissue using *mir*Vana^TM^ miRNA isolation kit (Ambion, Austin, TX, USA). Isolation was performed as per manufacturer’s instructions. The concentration of the total isolated RNA from the heart homogenate was measured immediately with NanoDrop^TM^ Lite Spectrophotometer (Thermo Scientific, Waltham, MA, USA). Total RNA was reverse transcribed to cDNA with TaqMan^®^ MicroRNA Reverse Transcription Kit (Applied Biosystems, Foster City, CA, USA) using T100^TM^ Thermal Cycler (Bio-Rad, Hercules, CA, USA) as per the manufacturer’s instructions. cDNA was amplified using the TaqMan^®^ Universal Master Mix II (Applied Biosystems, Foster City, CA, USA) with specific miRNA assays for miRNA-1 (Assay ID 002064), miRNA-133a (Assay ID 002246), miRNA-133b (Assay ID 002247), miRNA-135a (Assay ID 000460), and miRNA-29b (Assay ID 000413) (Applied Biosystems, Foster City, CA, USA). qPCR analysis was performed on CFX96^TM^ Real-Time System (Bio-Rad, Hercules, CA, USA), as per the manufacturer’s instructions. Amplification data were normalized to U6 snRNA (Assay ID 001973) (Applied Biosystems, Foster City, CA, USA).

### 2.6. Statistical Analysis

All data are expressed as mean ± SD and analyzed by one-way ANOVA with a Tukey’s multiple comparisons tests using GraphPad Prism v 7. Data distribution was assessed by Kolmogorov–Smirnov test, D’Agostino–Pearson omnibus normality test, and Shapiro–Wilk normality test. Statistical significance was accepted when *p* < 0.05.

## 3. Results

### 3.1. Dietary Fatty Acid Analysis

**Figure 1:**
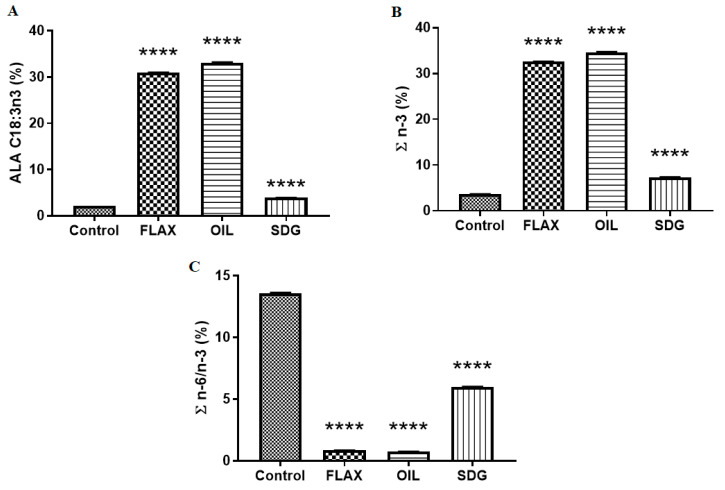
Effect of flaxseed supplementation in the diets. (**A**) ALA (C18:3n3) levels. (**B**) Sum of n-3 fatty acids (∑n-3) levels. (**C**) Ratio of n-6 and n-3 fatty acids (∑n-6/n-3) levels. Data are presented as mean ± SD, (*n* = 3). **** Significantly different from the control diet, *p* < 0.0001. ALA, alpha-linolenic acid; FLAX, chow diet supplemented with 10% ground flaxseed; OIL, chow diet supplemented with 4.4% ALA enriched flax oil; SDG, chow diet supplemented with 0.44% secoisolariciresinol diglucoside.

The macronutrient composition of the experimental diets has been reported previously [22]. The fatty acid profile of the diets is presented in Table 1. Notable differences were observed in the levels of saturated and monounsaturated fatty acids. As shown in Figure 1, due to the supplementation of the diets with flaxseed and flax oil, a significant increase of 14-fold and 15-fold, respectively, was observed in the levels of ALA (C18:3n3) as compared to the control diet. Similarly, total n-3 fatty acid levels were also significantly higher in the flaxseed supplemented diets when compared with the control diet, Figure 1. Consequently, the ratios of n-6 to n-3 fatty acid levels were significantly lowered in the flaxseed and the flax oil supplemented diets compared to the control diet, Figure 1. This increase in the levels of ALA, total n-3 fatty acid, and the ratios of n-6 to n-3 in the diet corresponds with an increase in the plasma levels of the same parameters observed in our previous study [22].

### 3.2. The Effect of Dietary Flaxseed and Its Components on the Expression of Collagen I

**Figure 2:**
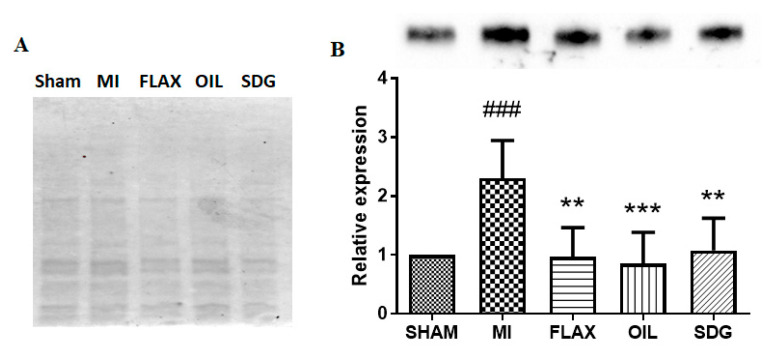
Effect of the dietary interventions on collagen I levels. (**A**), TGX gel visualization by Bio-Rad Gel Doc^TM^ XR+ imager to confirm equal protein loading. (**B**), Densitometric quantification of the immunoblots, data are presented as mean ± SD, *n* = 5–6. ^###^ Significantly different from sham, *p* < 0.001; ** significantly different from MI, *p* < 0.05; *** significantly different from MI, *p* < 0.001. ALA, alpha-linolenic acid; FLAX, chow diet supplemented with 10% ground flaxseed; MI, myocardial infarction; OIL, chow diet supplemented with 4.4% ALA enriched flax oil; SDG, chow diet supplemented with 0.44% secoisolariciresinol diglucoside.

A western blot analysis was performed to evaluate the expression levels of collagen protein. A significant >2-fold increase in the levels of collagen type I and alpha 1 (Col 1α1) protein was found in the untreated MI group compared to the sham control (Figure 2). Dietary supplementation with flax, flax oil, and SDG decreased the protein levels of Col 1α1 by 2.36, 2.69, and 2.13-fold, respectively, compared to the untreated MI group as shown in Figure 2.

### 3.3. The Expression of miR-133a Is Sensitive to the Flax Oil Treatment

**Figure 3:**
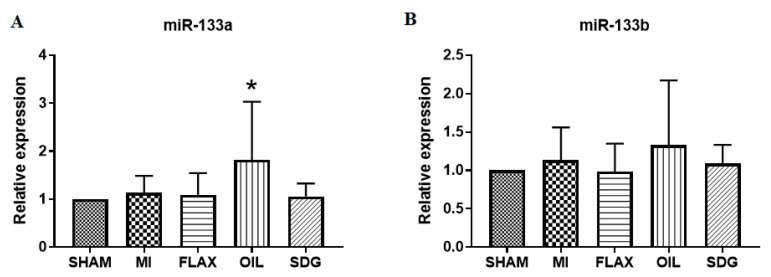
Effect of dietary interventions on miR-133 expression levels. (**A**) miR-133a; (**B**) miR-133b. Data are presented as mean ± SD, *n* = 5–6. * Significantly different from sham, *p* < 0.05. ALA, alpha-linolenic acid; FLAX, chow diet supplemented with 10% ground flaxseed; MI, myocardial infarction; OIL, chow diet supplemented with 4.4% ALA enriched flax oil; SDG, chow diet supplemented with 0.44% secoisolariciresinol diglucoside.

Dietary flaxseed supplementation was reported in our previous study to reduce myocardial infarct size and decrease the incidence of arrhythmias after an MI [22]. Downregulation of miR-133 expression after an MI is related to the death of cardiomyocytes as well as the development of ventricular fibrillation. In this study, the expression of miR-133a and miR-133b was analyzed in the postinfarct cardiac tissue after 8 weeks of CAL surgery. No significant differences in the expression patterns of both the isoforms of miR-133 were observed in the untreated MI group when compared to the sham control (Figure 3). However, flax oil treatment significantly upregulated the expression of miR-133a compared to the control tissue. Interestingly, miR-133b expression was not sensitive to the flax oil treatment.

### 3.4. Upregulation of miR-135a and -29b by the Flax Oil Supplementation

**Figure 4:**
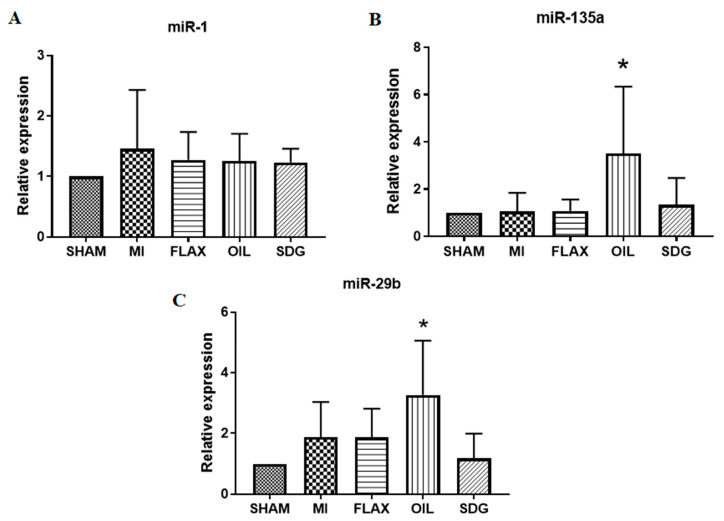
Effect of dietary interventions on: (**A**) miR-1; (**B**) miR-135a; (**C**) miR-29b, expression levels. Data are presented as mean ± SD, *n* = 5–6. * Significantly different from sham, *p* < 0.05. ALA, alpha-linolenic acid; FLAX, chow diet supplemented with 10% ground flaxseed; MI, myocardial infarction; OIL, chow diet supplemented with 4.4% ALA enriched flax oil; SDG, chow diet supplemented with 0.44% secoisolariciresinol diglucoside.

Increased expression of miR-1 is arrhythmogenic in the ischemic hearts, whereas the downregulation of miR-135a and miR-29b is associated with apoptosis of cardiomyocytes and extensive cardiac fibrosis, respectively. The flax oil supplementation significantly upregulated the expression of miR-135a and miR-29b in the postinfarct cardiac tissue 8 weeks after CAL surgery (Figure 4). An approximately 3.5-fold and 3.3-fold increase in the expression levels of miR-135a and miR-29b, respectively, was observed with respect to the control group, as shown in Figure 4. No such changes were observed in the expression levels of miR-1 amongst experimental groups.

### 3.5. The Effect of Dietary Flaxseed and Its Components on the Expression of NCX

**Figure 5:**
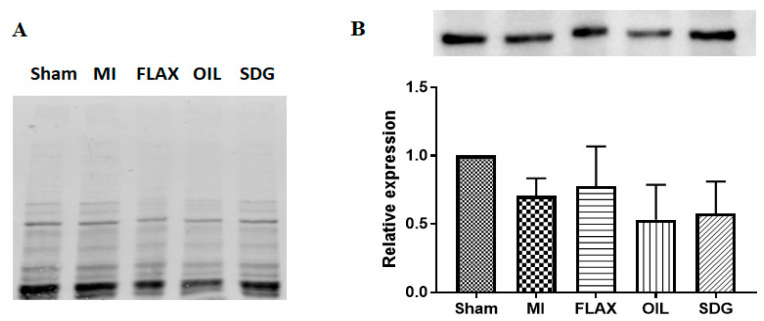
Effect of the dietary interventions on NCX levels. (**A**) TGX gel visualization by Bio-Rad Gel Doc^TM^ XR+ imager to confirm equal protein loading. (**B**) Densitometric quantification of the immunoblots, data are presented as mean ± SD, *n* = 5. ALA, alpha-linolenic acid; FLAX, chow diet supplemented with 10% ground flaxseed; MI, myocardial infarction; OIL, chow diet supplemented with 4.4% ALA enriched flax oil; SDG, chow diet supplemented with 0.44% secoisolariciresinol diglucoside.

A western blot analysis was performed to evaluate the expression levels of NCX protein. No changes were observed in the expression levels of NCX between experimental groups as shown in Figure 5.

## 4. Discussion

Dietary supplementation with flaxseed before and after an MI has been reported to decrease the incidence of arrhythmias, reduce the myocardial infarct size, decrease cardiac fibrosis, exert an anti-inflammatory effect, and prevent excessive left ventricular dilatation [22]. The present study was thus undertaken to: (a) investigate the effect of flaxseed on modulating the expression levels of miR-1, miR-133a, miR-133b, miR-135a, and miR-29b involved in adverse cardiac remodeling, and, (b) identify the potential bioactive compounds within flaxseed that may be responsible for the observed effects. In the current study, the data demonstrate that in an animal model of acute MI induced by coronary artery ligation, dietary supplementation with flaxseed regulates the expression of miR-133a, miR-135a, miR-29b, and collagen I.

miRNAs are short, non-coding nucleotides that negatively regulate the expression of target genes by mRNA degradation or translational repression. Altered expression profiles of miRNAs have been associated with various cardiovascular diseases including arrhythmias, MI, and heart failure [25,26]. Notably, an essential role of miRNAs has been reported in several pathological processes of ischemia-induced cardiac damage including cardiac fibrosis and even the death of cardiomyocytes [15,27]. Various nutritional components can modulate the expression of diverse miRNAs [3]. Omega-3 PUFAs, for example, protect cardiomyocytes after an MI by upregulating the anti-apoptotic miRNAs and downregulating the pro-apoptotic miRNAs [28,29]. Flaxseed is the richest source of the plant-based omega-3 PUFA, ALA. Dietary supplementation with flaxseed has demonstrated anti-arrhythmic, antiatherogenic, antihypertensive, and cholesterol-lowering effects in animal and human studies [21]. Flaxseed and its components modulate the expression of miRNAs in breast cancer cells [30,31] and irradiated lung tissue [32]. However, no study has examined the effect of flaxseed or ALA on miRNAs in the setting of an MI.

miR-133 is expressed in cardiomyocytes and cardiac fibroblasts [33]. miR-133, which is frequently associated with an MI, has two isoforms -133a and -133b [10]. Our data showed that the expression of miR-133a but not miR-133b was upregulated by dietary flaxseed oil supplementation in rat hearts 8 weeks after coronary artery ligation. The location of miR-133a and miR-133b on separate chromosomes may explain why only one of the isoforms was upregulated [34]. Overexpression of miR-133 reduces the apoptosis of cardiomyocytes [12]. Pro-apoptotic genes such as DAPK2, APAF1, caspase-9, Bcl-2, and BMF have been identified as the target of miR-133 [10]. Interestingly, miR 133a also improved cardiac reprogramming to produce beating cardiomyocytes from murine and human fibroblasts by repressing Snai1, which regulates epithelial to mesenchymal transition [14]. This is important because cardiomyocytes are unable to replicate and regenerate any lost contractile tissue after an MI [35]. By upregulating the expression of cardiogenic miR-133, dietary flaxseed may be a viable strategy to promote cardiac repair.

Our results also demonstrated that flaxseed oil treatment upregulated the expression of miR-135a and miR-29b after an MI. The upregulation of miR-135a has previously reduced MI size and exerted an anti-apoptotic and anti-fibrotic effect in a model of ischemia-reperfusion injury [18]. miR-29b is highly expressed in cardiofibroblasts and is reported to target numerous genes involved in extracellular matrix production and fibrosis such as collagen isoforms, elastin and matrix metalloproteinases [4,15,36]. An upregulation of miR-135a and miR-29b expression results in decreased myocardial fibrosis [16,17,18]. Increased accumulation of fibrillar collagen in the cardiac matrix is a hallmark of myocardial fibrosis [37]. Type I collagen is the most abundant as it accounts for 85% of the collagen content within the myocardium [38]. Previous work from our lab has shown increased histochemical staining after an MI induced by coronary artery ligation as evidence of increased myocardial fibrosis [22]. The tissue fibrosis was prevented when flaxseed and flaxseed oil was included in the diet before and after the MI was induced [22]. Consistent with these findings, an increased expression of collagen I was detected in the present study after the MI which was normalized when dietary flaxseed oil was included in the diet of the rats. These effects on myocardial fibrosis by miR-29b and miR-135a are complemented by the actions of miR-133. miR-133 inhibits the expression of collagen 1A1 and connective tissue growth factor (CTGF) to exert its anti-fibrotic effect [39]. During overexpression of miR-133, inflammatory cell infiltration in the myocardium is reduced after an MI [11]. Taken together, upregulation of miR-29b, miR-135b and miR-133a and the subsequent decrease in collagen I levels by flaxseed oil may represent the primary mechanisms through which the ALA content of flaxseed exhibits its anti-fibrotic effect.

The anti-arrhythmic action of dietary flaxseed oil may also be associated with its capacity to alter miRNA expression. The downregulation of miR-133 has been related to the incidences of ventricular fibrillation in MI patients [13]. miR-133 can decrease the expression of hyperpolarization-activated cyclic nucleotide-gated ion channel 2 (HCN2), which determines cardiac automaticity [13]. Alternatively, miR-135a can modulate the expression of the thioredoxin-interacting protein (TXNIP), protein tyrosine phosphatase 1B (PTP1B) protein, and the transient receptor potential melastatin 7 (TRPM7) channel [8,18]. miR-135a also targets the sodium-calcium exchanger type 1 (NCX1) [19]. NCX1 activity can be arrhythmogenic causing early- and delayed-after depolarizations [19]. The expression and function of NCX can change following a myocardial infarction [40]. We have previously demonstrated that ALA can inhibit NCX activity [41]. However, in the present study no alterations in the expression levels of NCX in any of the experimental groups was noted. Although NCX is one of the prime targets of miR-135a, the neutral effect could be due to a lack of change in the expression levels of NCX in MI groups 8 weeks after surgery compared to the sham group in our study. Increased expression of miR-135a can reduce the spontaneous beating frequency of rat neonatal cardiomyocytes [19]. Previous work from our lab has shown dietary flaxseed and flaxseed oil is anti-arrhythmic in models of ischemia-reperfusion injury to the heart [42] and coronary artery ligation-induced MI. Flaxseed was particularly effective against ventricular fibrillation (VF) [42] and decreased expression of miR-133 increases the incidence of VF [13]. The capacity of dietary flaxseed oil to induce the expression of miR-133 and miR-135a may, therefore, represent the anti-arrhythmic mechanism of dietary flaxseed oil. Overall, these studies suggest that the reduced MI size, fibrosis, inflammation and incidence of arrhythmias shown previously [22] may have been associated with the ALA content of dietary flaxseed.

While the ALA component of flaxseed is highly likely to have contributed to the observed cardioprotective effect in this study, the role of other potential agents cannot be ruled out. Flaxseed could have produced its effect through the long-chain PUFA metabolites of ALA, eicosapentaenoic acid (EPA) and docosahexaenoic acid (DHA). EPA and DHA have been reported to modulate the expression of miRNAs involved in inflammation [43]. PUFAs in flaxseed may also have indirectly mediated their effect through the specialized proresolvins lipid mediators such as resolvins, maresins, protectins, and lipoxins that contribute to anti-inflammatory effects via miRNAs [44]. It is important to note that limited studies have examined the link between n-3 PUFAs and miRNAs. Therefore, it is essential that further investigations with miRNA mimickers and inhibitors are carried out to understand the mechanism behind the effect of n-3 PUFAs on specific miRNAs.

It is difficult to know for certain why the flax oil group alone upregulated the expression of certain miRNA and not ground flax despite having the same proportion of ALA in both groups. However, we have shown previously that the ALA provided in flax oil is more bioavailable compared to the ALA in ground flax [45]. The ALA that is presented in oil form likely helps to emulsify the ALA and promote its transport across membranes, whereas the high fiber content in ground flaxseed would have the opposite effect. Therefore, the higher plasma levels of ALA achieved through flax oil ingestion may have contributed to changes in the miRNA expression. The amount of ALA bioavailable from ground flax was likely insufficient to induce any change. It is unlikely that the SDG in the flaxseed had an inhibitory/differential effect on the flax ALA’s action on miRNA expression because SDG did not affect miRNA values when tested on its own.

The data presented in this study were analyzed from viable tissue 8 weeks post-MI. We had to do this to ensure a stable recovery period. The post-infarct healing period is considered complete after 6-8 weeks following MI as the infarct area becomes inert tissue [46]. However, a differentially regulated miRNA profile is known to occur at different phases of cardiac healing. Changes in the expression levels of miRNAs in accordance with the progression of the MI were reported in a rat model of AMI at day 3, 7, and 14 post-MI [47]. Another study confirmed dramatic changes in miRNA expression acutely following MI (2 days), but these changes tended to normalize over time as the myocardium healed [48]. It is possible that the specific miRNAs analyzed in our study had normalized over 8 weeks post-MI. The results we observed, therefore, may be a significant under-representation of the changes that occurred soon after the induction of the MI. The miRNAs that were statistically significantly upregulated by flax oil in comparison to the sham control and only tended to be upregulated (but not statistically significant) in comparison to the MI group would suggest that these miRNAs are sensitive to ALA and may have been more highly upregulated at an earlier time point.

Although our study has generated data supporting the positive effect of flaxseed on miRNAs, certain limitations are to be acknowledged. One concern is the effect of flax and its components during early days of cardiac repair following an MI. However, the sensitivity of miR-133a, miR-135a, and miR-29b to the ALA-rich flax oil treatment 8 weeks post-MI strongly suggest that dietary flaxseed will exert a favorable role on miRNAs during acute ischemic insult. Previous studies exploring pharmacokinetics of miR-mimics have demonstrated that levels of miR-mimics remain elevated only for a few days after delivery [49], and usually return to the baseline value a week after injection [50]. Thus, the fact that certain miRNAs remained slightly upregulated 8 weeks post-MI implies that it may be possible to maintain therapeutic regulation of miRNAs in MI patients by flaxseed consumption. It should also be noted as a limitation that this study could not examine target genes/proteins of the related miRNAs because of limited availability of tissues. Future studies are strongly warranted to verify the findings using miR-inhibitors and downstream target analysis to validate the association between ALA and responsive miRNAs. Among other concerns include assessing the effect of dietary flaxseed on circulating miRNAs levels that could be of more clinical relevance diagnostically.

## 5. Conclusions

**Figure 6:**
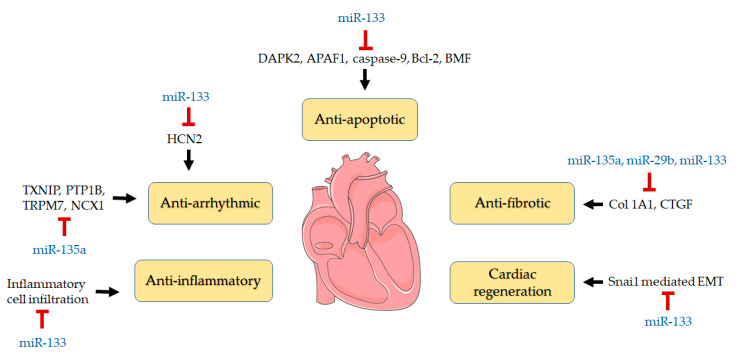
ALA-rich flaxseed oil upregulates the expression of miR-133a, miR-135a, and miR-29b and consequently exerts cardioprotective effect.

In summary, this study has provided the first evidence that miR-133a, miR-135a, and miR-29b are sensitive to dietary flaxseed oil and associated with significant cardioprotection in a model of MI (Figure 6). These findings reflect a selective change in miRNA expression since miR-1 and miR-133b were unaffected during the course of this investigation. The present study also suggests the cardioprotective effect of flaxseed in the setting of an acute MI is likely mediated, at least in part, via its ALA content (Figure 5). These data complement previous studies on hypertension and atherosclerosis that have also attributed the health benefits of dietary flaxseed to its content of ALA [24,51,52,53]. Overall, the experimental evidence generated in this study suggests that dietary flaxseed can modulate the expression of certain miRs involved in adverse cardiac remodeling to produce a favorable effect in an MI.

APAF1, apoptotic protease activating factor 1; Bcl-2, B-cell lymphoma 2; BMF, Bcl-2 modifying factor; Col1A1, collagen, type 1, alpha 1; CTGF, connective tissue growth factor; DAPK2, death-associated protein kinase 2; EMT, endothelial-mesenchymal transition; HCN2, potassium/sodium hyperpolarization-activated cyclic nucleotide-gated ion channel 2; NCX1, sodium calcium exchanger; PTP1B, protein tyrosine phosphatase 1B; TRPM7, transient receptor potential ion channel subfamily M, member 7; TXNIP, Thioredoxin-interacting protein.

## Figures and Tables

**Table 1:** Dietary fatty acid analysis.

Fatty Acid	CONTROL	FLAX	OIL	SDG
**C10:0**	t	t	t	t
**C12:0**	0.01 ± 0.00	0.01 ± 0.00	0.01 ± 0.00	0.02 ± 0.00 *
**C14:0**	0.49 ± 0.00	0.51 ± 0.01	0.50 ± 0.00	1.06 ± 0.00 *
**C15:0**	0.07 ± 0.00 *	0.08 ± 0.00 *	0.08 ± 0.00 *	0.16 ± 0.00 *
**C16:0**	13.67 ± 0.02 *	10.17 ± 0.02 *	10.16 ± 0.02 *	16.37 ± 0.01 *
**C17:0**	0.12 ± 0.00	0.12± 0.00 ^‡^	0.12 ± 0.00 *	0.21 ± 0.00 *
**C18:0**	1.92 ± 0.00	3.00 ± 0.00 *	4.10 ± 0.00 *	2.37 ± 0.00 *
**C20:0**	0.32 ± 0.00	0.19 ± 0.00 *	0.21 ± 0.00 *	0.22 ± 0.00 *
**C22:0**	0.17 ± 0.00	0.18 ± 0.00 *	0.19 ± 0.00 *	0.22 ± 0.00 *
**C24:0**	0.19 ± 0.00	0.17 ± 0.00 ^‡^	0.15 ± 0.00 *	0.21 ± 0.00
**Total SFA**	16.98 ± 0.02	14.43 ± 0.02 *	15.51 ± 0.02 *	20.82 ± 0.01 *
**C14:1**	t	t	t	t
**C16:1t**	0.05 ± 0.00	0.05 ± 0.00 ^‡^	0.04 ± 0.00 *	0.07 ± 0.00 *
**C16:1**	0.68 ± 0.01	0.66 ± 0.01	0.60 ± 0.01 ^‡^	1.33 ± 0.02 *
**C17:1**	4.19 ± 0.03	4.16 ± 0.01	3.93 ± 0.02 *	9.57 ± 0.06 *
**C18:1**	23.16 ± 0.01	19.53 ± 0.01 *	18.43 ± 0.01 *	15.15 ± 0.01 *
**C18:1n7c**	0.81 ± 0.00	0.87 ± 0.00 *	0.83 ± 0.00 ^‡^	1.19 ± 0.01 *
**C20:1**	0.39 ± 0.00	0.33 ± 0.00 *	0.25 ± 0.01 *	0.50 ± 0.01 *
**C22:1**	0.03 ± 0.00	0.03 ± 0.00	0.04 ± 0.00 *	0.05 ± 0.00 *
**C24:1**	0.05 ± 0.00	0.04 ± 0.00	0.04 ± 0.00	0.10 ± 0.00 *
**Total MUFA**	29.36 ± 0.03	25.66 ± 0.01 *	24.16 ± 0.01 *	27.95 ± 0.05 *
**C18:2**	49.82 ± 0.04	27.13 ± 0.03 *	25.55 ± 0.06 *	43.53 ± 0.03 *
**C18:3n6**	0.03 ± 0.00	0.03 ± 0.00	0.03 ± 0.00	0.06 ± 0.00 *
**C20:2**	0.04 ± 0.00	0.05 ± 0.00 ^‡^	0.04 ± 0.00	0.08 ± 0.00 *
**C20:3n6**	0.01 ± 0.00	0.01 ± 0.00	0.01 ± 0.00	0.01 ± 0.00
**C20:4**	0.06 ± 0.00	0.08 ± 0.00 *	0.07 ± 0.00 *	0.16 ± 0.00 *
**C20:3n3**	0.00 ± 0.00	0.01 ± 0.00 *	0.01 ± 0.00 *	0.00 ± 0.00
**C20:5**	0.75 ± 0.00	0.77 ± 0.00	0.72 ± 0.00	1.65 ± 0.01 *
**C22:2**	t	t	t	t
**C22:4**	0.01 ± 0.00	0.01 ± 0.00	0.01 ± 0.00	0.02 ± 0.00 *
**C22:5n6**	0.01 ± 0.00	0.02 ± 0.00 *	0.01 ± 0.00	0.04 ± 0.00 *
**C22:5n3**	0.09 ± 0.00	0.10 ± 0.00 *	0.09 ± 0.00	0.21 ± 0.00 *
**C22:6n3**	0.70 ± 0.00	0.75 ± 0.00 *	0.70 ± 0.00	1.58 ± 0.01 *
**Total PUFA**	53.66 ± 0.04	59.91 ± 0.02 *	60.33 ± 0.02 *	51.23 ± 0.04 *

Data are presented as mean ± SD, *n* = 5–6. ^‡^ Significantly different from the control diet, *p* < 0.05; * Significantly different from the control diet, *p* < 0.001. SFA, saturated fatty acid; MUFA, monounsaturated fatty acid; PUFA, polyunsaturated fatty acid; t, trace. FLAX, chow diet supplemented with 10% ground flaxseed; OIL, chow diet supplemented with 4.4% ALA enriched flax oil; SDG, chow diet supplemented with 0.44% secoisolariciresinol diglucoside.
